# The Use of Direct-to-Consumer (DTC) Pharmaceutical Advertisements in Televised and Print Formats as a Teaching Tool in a Pharmacy Curriculum

**DOI:** 10.3390/pharmacy9030149

**Published:** 2021-09-02

**Authors:** Asha Suryanarayanan

**Affiliations:** Department of Pharmaceutical Sciences, Philadelphia College of Pharmacy, University of the Sciences, Philadelphia, PA 19104, USA; a.suryanarayanan@usciences.edu; Tel.: +1-215-596-7152

**Keywords:** direct to consumer (DTC), drug advertisements, pharmacology, survey

## Abstract

The overall goal of this study was to employ direct-to-consumer advertisements (DTCAs) as a teaching tool in a Doctor of Pharmacy (PharmD) curriculum. The objectives of this pilot study were to investigate the following questions: 1. Do DTCAs generate student curiosity about the advertised drug and associated disease? 2. Can DTCAs help students understand and reinforce various pharmacological aspects of the drug? 3. How do students perceive DTCAs? A DTCA-based teaching tool was employed in a pharmacology course taken by P2 (second professional year) PharmD and final year (U4) Bachelor of Science (BS) in Pharmacology–Toxicology students. A voluntary online survey was administered to students to determine the effectiveness of this tool. Survey data were analyzed quantitatively and qualitatively. 70–85% of responding students indicated that this teaching tool was an effective visual aid for learning pharmacology and correlating the drug to disease state, mechanism of action, and adverse effects. Moreover, themes identified from the qualitative analysis suggest that this teaching tool may be useful to enhance patient counseling skills in students. The initial implementation of this DTCA-based teaching tool proved to be successful, and a similar approach can be easily implemented in other pharmacotherapy and laboratory courses. Further studies are needed to determine if this approach can improve patient counseling skills.

## 1. Introduction

Advertisements (ads) or commercials in various formats such as televised, print, and radio touch our lives every day. Among various TV commercials, direct-to-consumer (DTC) televised pharmaceutical drug advertisements (DTCAs) are usually short clips that quickly mention the drug’s brand name and what the drug is used for, and usually finish with a list of the drug’s adverse effects and contraindications. DTCAs in the Unites States began to appear in print in the early 1980s and quickly spread to broadcast formats [1]. Aside from New Zealand, the United States is the only country that allows DTC prescription medication ads to be aired on television [2]. Now, DTCAs are distributed via television, radio, newspapers, magazines, and—increasingly—the social media and internet (labeled eDTCA) [2]. Many pharmaceutical companies have a dedicated social media website and utilize platforms such as Facebook, Twitter, etc. for eDTCA [3]. Social media eDTCA typically consists of help-seeking advertisements using either text and/or video [4]. While the Food and Drug Administration (FDA) regulates DTCAs disseminated via traditional media, the rapid growth of eDTCA presents several regulatory challenges [5]. Nevertheless, both patients and health care providers are exposed to DTCA in various forms.

While most televised drug commercials are focused on marketing prescription medications, some commercials are also focused on common over-the-counter (OTC) medications such as Tylenol^®^ and aspirin. In contrast to prescription drug advertisements that are regulated by the FDA, the Federal Trade Commission (FTC) is responsible for regulating OTC drug ads. While some televised drug commercials are perceived as memorable, funny, and persuasive, some are perceived as annoying. Nonetheless, the number of televised and print pharmaceutical drug commercials is on the rise, and pharmaceutical companies pour billions of dollars toward drug promotion via DTCAs [6]. Research suggests that DTCAs can either be detrimental or beneficial to public health [7]. While supporters of DTCAs argue that drug commercials make the patient more well informed, opponents argue that DTCAs do not make a significant impact on health benefits and may mislead patients and increase prescribing costs [8].

It is estimated that around 40–50% of patients seek further drug information from any information source after DTCA exposure [9]. Prior research indicates that physicians are the most common information sources for patients, pharmacists are the second most common source, and the internet is the fastest-growing information source [9]. A recent review of the literature suggests that patients tend to prefer the internet for the ease of access to information, while they trust their physicians and pharmacists more for their clinical expertise and experience [10,11]. Thus, pharmacy students and licensed pharmacists have pivotal roles in answering patients’ questions about advertised drugs. Therefore, awareness about information presented in DTCAs may help pharmacy students and pharmacists answer such potential questions, appreciate any misleading or false claims in DTCAs, and have informed discussions with their patients. This awareness about DTCAs and appropriate patient counseling about DTCAs is especially important in current times due to growing concern that online promotional activities via eDTCA can negatively impact public health and patient safety.

A previous study assessed if pharmacy students were aware of the quality and extent of information provided in DTCAs [12]. The authors of this study concluded that pharmacy students’ knowledge about DTCA regulations needs improvement and further suggested that DTCAs should be further used in pharmacy curricula to increase pharmacy students’ awareness about DTCAs. However, to date, DTCAs have remained largely unexplored as a pedagogical tool in pharmacy and other health professions curricula.

The overall goal of the current pilot study was to develop and evaluate a DTCA-based (both print and televised ads) teaching tool in a pharmacy curriculum. An online survey of various pedagogical approaches shows that advertisements, in general, have been used as an effective teaching tool in several English as a second language (ESL) courses as well as in psychology [13,14]. However, to this instructor’s knowledge, DTCA has not been employed as a teaching tool/approach in the classroom to teach concepts in pathophysiology or pharmacology. This instructor developed a DTCA-based teaching approach in a pharmacy practice lab in the integrated PharmD curriculum at her earlier place of employment as an Assistant Professor at the Manchester University College of Pharmacy, Fort Wayne, Indiana. Course evaluation results indicated that the laboratory exercise was received favorably by the students in Indiana. In the current pilot study, a DTCA-based teaching approach was developed and employed in a Pharmacology course in the PharmD program at the Philadelphia College of Pharmacy, University of the Sciences, Philadelphia. Survey data gathered at the Philadelphia College of Pharmacy are presented here. The objectives of this study were to determine the following: 1. Do DTCAs generate student curiosity about the advertised drug (or drug class) and the associated disease area? 2. Do DTCAs help the students understand and reinforce various pharmacological aspects of the drug, such as the mechanism of action, adverse effects, and contraindications? 3. How do students perceive DTCAs?

## 2. Materials and Methods

PC412-440-740 is a four-credit course (4 credits = 4 h class time per week in a 16-week semester) taken by PharmD students in the second professional year (P2), final year BS in Pharmacology–Toxicology (Pharm–Tox) undergraduate (U4) students, as well as graduate students, at the Philadelphia College of Pharmacy, University of the Sciences. The PharmD and BS in Pharm–Tox programs are 4 years in duration. This course is a team-taught course that focuses on the pharmacology of drugs acting on the autonomic nervous system, cardiovascular, endocrine, and central nervous systems. This course is taught by three pharmacology faculty members and serves as a foundational pharmacology course for P2 students before students enroll in pharmacotherapeutics-based course work in the P3 year. A DTCA-based teaching tool was employed to better communicate complex pharmacology concepts to a cohort of 211 students in the PC412-440-740 course. This specific cohort of students included 201 P2 year PharmD students and 10 U4 Pharm–Tox undergraduate students. No graduate students were enrolled in this particular cohort. P2 PharmD and U4 Pharm–Tox undergraduate students attended the lectures together and took the same assessments in the course.

### 2.1. Study Design and Participants

The Institutional Review Board at the University of Sciences approved this study (ID# 1709661-1, 11 February 2021). A total of 211 students were enrolled in the PC412-440-740 pharmacology course wherein the teaching aid was employed. These students included P2 PharmD students (*n* = 201) and U4 students in the BS Pharmacology–Toxicology program (*n* = 10) in the College of Pharmacy. Students taking this course were exposed to hundreds of new drugs that have diverse indications, mechanisms of action, adverse effects, and contraindications. The DTCA-based teaching approach was applied in the autonomic nervous system (ANS) and central nervous system (CNS) blocks of the course. Throughout the ANS and CNS blocks, various drug commercials (print and televised versions) were employed in the classroom. The commercial was chosen based on the disease state and drug class that was focused on. For example, the DTCA for Lunesta^®^ (eszopiclone) was played before discussing insomnia and sedatives such as Z-drugs (insomnia medications such as eszopiclone (Lunesta^®^), zaleplon (Sonata^®^) and zolpidem (Ambien^®^, Edluar^®^, and Zolpimist^®^) are sometimes referred to as “Z-drugs”). More examples are shown in Table 1. Before playing/showing the commercial, an announcement was made to students stating that the instructor had no vested interest in the pharmaceutical company or product marketed in the DTCA. These commercials were first employed as an introduction before discussing the disease state and the associated drug class. Following the commercial, the drug or drug class was discussed in detail. Key symptoms/statements from the advertisement such as adverse effects and contraindications were discussed as applicable. After a detailed discussion about the drug, the same advertisement was revisited again. Students were asked to reflect upon key statements about the drugs’ adverse effects, warnings, and contraindications mentioned in the DTCA and their understanding of the associated pharmacological mechanisms. Videos for commercials were obtained from www.ispot.tv (accessed on March 2021) or YouTube (List of links provided in Flomax^®^: https://www.youtube.com/watch?v=hs25VBxFFRg Epipen^®^: http://www.ispot.tv/ad/7tD4/mylan-summer-camp (accessed on March 2021). Bayer low dose Aspirin: http://www.ispot.tv/ad/7XzC/bayer-low-dose-crossword-puzzle (accessed on March 2021). Detrol^®^: https://www.youtube.com/watch?v=GNz7_31Ei_g (accessed on March 2021). Thorazine^®^ print ad: http://www.biopsychiatry.com/chlorpromazine/thorazine.html (accessed in March 2021).).

### 2.2. Data Collection

DTCA commercials were employed in the classroom along with follow-up clicker questions and discussion. In order to assess this pilot DTCA-based teaching approach, a voluntary, online survey was administered to the students. Survey monkey^®^ (San Mateo, CA, USA) was employed to administer the survey. Students were not provided any extra credit for taking this assignment. The survey link was only available to students enrolled in PC412-440-740. Each student could only take the survey once, and information such as name, student ID, etc. was not collected.

The survey had six questions to gauge if the drug commercial stimulated student interest, was helpful in correlating the drug to the disease state, etc. Table 2 has a list of questions. Of the six questions, five employed the Likert scale: 1 = strongly disagree to 6 = strongly agree. The sixth question was a choice between three drug advertisements employed in the autonomic nervous system block. The questions and quantified student responses are included in Table 2. The themes identified and select student comments are shown in Table 3.

### 2.3. Data Analysis

Student survey responses were copied verbatim to Microsoft Excel (2019) for further analysis. Qualitative comments were reviewed to identify major themes and representative quotes. Descriptive statistics were used to summarize quantitative survey results.

## 3. Results

Of the 211 students enrolled in the PC412-440-740 pharmacology course, 100 students voluntarily took this anonymous survey, yielding a survey rate of ~47%. The 100 students that completed the survey comprised 90 PharmD students and 10 U4 Pharm–Tox undergraduate students. Overall, the survey results (Table 2) indicate that the majority of students (~67–80%) agree or strongly agree that watching a drug commercial before learning about the pharmacology of the drug stimulated their interest in the drug (78%, *n* = 78), stimulated their curiosity about the drug’s mechanism of action and therapeutic uses (67%, *n* = 67) and helped correlate the drug with the corresponding disease state (87%, *n* = 87). Most students also recommended that drug commercials should be continued as a teaching tool in this and other courses (69%, *n* = 69). Some students said that humor and catchy lines in video advertisements helped them correlate information learned in class (*n* = 15) and also helped with recalling information during exams (*n* = 41).

In order to gain insight into student perception, the last survey question asked an open-ended question about how students perceived DTCAs. Major themes gathered by qualitative analysis of survey results are shown in Table 3. Representative comments for each theme are also included in Table 3.

## 4. Discussion

Advertisements for OTC and prescription medications are frequently seen in print and televised formats. As such, DTCA represents a source of information for existing and prospective patients. A survey of the literature indicates that drug advertisements have not been widely explored as a pedagogical tool in pharmacy programs. It was hypothesized that DTCA would be a useful tool to enhance learning and student engagement and to improve patient-counseling skills. Specifically, a pilot survey was conducted in a P2 year Pharmacology course taken by students in the PharmD and BS in pharmaceutical programs (U4). Overall, 70–85% of the students that took the survey thought that this teaching tool was an effective visual aid for learning pharmacology and correlating the drug to disease state, mechanism of action, and adverse effects. In class, the instructor noted that several students paid attention to portions of the DTCA videos that covered potential adverse effects and contraindications. The students appeared visually interested and engaged in the material taught. Additionally, students also commented that pharmacists can play their part in clearing any misconceptions and questions that potential patients may have about a drug after watching a DTC advertisement. Students also commented that after this classroom experience, they pay close attention to the drug’s side effects and are critical of certain aspects of the commercial such as the speed of the commercial, warnings about adverse effects, contraindications, etc.

Students that responded to the survey also recommended that the DTCA-based teaching approach should be continued in the PharmD curriculum. This approach can be easily extended to other PharmD courses. This approach creates valuable opportunities to design laboratory exercises to mimic patient encounters and improve patient counseling skills. For example, students can be asked to counsel a standardized patient who has watched a DTCA for a particular drug and has questions/concerns about the side effects of the drug they have been prescribed. Alternatively, the standardized patient may also ask the student pharmacist if they need the advertised drug based on their self-diagnosis. This DTCA-based tool can also be valuable in team-based student presentations, where each team is assigned or allowed to select a DTCA. For example, students can develop a presentation based on the drug and present it to their peers and faculty. Students can also critique the advertisement about any potential missed information, misinformation, etc. Their peers in the audience can then ask questions and provide feedback. This approach can be valuable in understanding and retaining key concepts in pharmacology, which is sometimes perceived as a “dry” subject by students. Moreover, advertisements for drugs such as Thorazine^®^ also added a historic and social perspective, where the students learned about the stigma associated with psychiatric disorders and how these disorders were perceived differently by society several years ago.

Instructors need to use choose DTCA examples judiciously based on factors such as drug class and disease area discussed in the course, available class time, etc. This DTCA-based teaching aid was also employed in other online pharmacology courses at the University of the Sciences during the COVID-19 campus closure. Informal student feedback indicated that DTCA represented a great attention grabber and kept them engaged in the online classroom. Thus, this approach can be easily applied in both in-person and online teaching. The survey response rate in this study was 47%. Although non-response bias cannot be ruled out from this study, it is important to note that this pilot survey was entirely anonymous and voluntary—there was no incentive offered to students for completion of the survey. In future studies, students will be offered more time to complete the survey(s) and a few reminders will also be sent to non-respondents.

Going forward, a pre- and post-survey will be used to assess changes in student knowledge, confidence, etc. to further assess this teaching tool. We will also assess if there is any correlation between the students’ perceptions and their grades earned in course assessments. The effectiveness of this approach will be evaluated in multiple cohorts of students. This approach is easily applicable to integrated pharmacotherapeutics courses in the new competency-driven curriculum employed by the college, as well as other schools of pharmacy. Although DTCAs are not widely employed outside the US and New Zealand, various DTCA videos and print ads are readily available on the internet (see Section 2.1 for examples of video links). Thus, these DTCA sources can be used as pedagogical tools even in countries where DTCA is not commonly employed. In addition, DTCA-based exercises focused on public health aspects such as risk factors for disease, disease prevention, etc. can also be developed for pharmacy students, as well as students in other health care professions [15].

## 5. Conclusions

This pilot study successfully evaluated the utility of DTCA as a teaching tool in a pharmacy curriculum. A majority of students surveyed indicated that this DTCA-based teaching aid helped increase their curiosity about drug pharmacology, correlate drugs with disease states, and retain information. Student comments also indicated that this teaching aid creates new opportunities for future presentation-based assessments and exercises designed to improve patient counseling. Further studies are necessary to evaluate the effectiveness of this DTCA-based teaching approach in other pharmacotherapy courses and professional laboratory-based assignments.

## Figures and Tables

**Table 1:** Examples of drug advertisements employed.

Drug Name (Brand and Generic)	Drug Class/Indication	Commercial
Detrol^®^ tolterodine	Anticholinergic used to control overactive bladder	1. The TV commercial shows a traffic police offer with an overactive bladder saying “Gotta go right now.” 2. A print advertisement displaying many toilets was shown.
Epipen^®^ epinephrine	Autoinjector used in several life-threatening allergies	TV commercial shows several children going to a summer camp.
Flomax^®^ tamsulosin	Alpha1 blocker used in treatment of benign prostatic hyperplasia	TV commercial showing older men playing golf.
Bayer aspirin^®^ aspirin	Low-dose aspirin to prevent heart attacks	TV commercial showing an older man solving a crossword that said “Your heart attack happens today.”
Lunesta^®^ eszopiclone	Non-benzodiazepine sedative	TV commercial shows a glowing ladybug going into restless people’s windows and guiding them to sleep.
Thorazine^®^ chlorpromazine	Older class of antipsychotic medication	An old black and white print advertisement stating “When the patient lashes out against ‘them’-Thorazine ^®^ quickly puts an end to his violent outburst.

**Table 2:** Survey questions (*n* = 100 students).

Survey Question	Agreement *n* (%) ^a^	Neutral *n* (%)	Disagreement *n* (%) ^b^
Watching a drug commercial before learning about the pharmacology of the drug stimulated your interest in the drug.	78 (78)	19 (19)	3(3)
Viewing a drug commercial stimulated your curiosity about the mechanism of action and therapeutic uses of the drug.	67 (67)	29 (29)	4 (4)
Viewing a drug commercial was helpful in correlating the drug with the disease state that drug is used for.	87 (87)	11 (11)	2 (2)
As a student, you would recommend that drug commercials be continued to be used as a teaching tool in PC412-440-740 and other courses.	69 (69)	20 (20)	1(1)
Open ended survey questions:			
Question		Responses	
In the autonomic nervous system pharmacology section of the P412-440 course, the following commercials were played in class: Detrol, Flomax, Epipen. Which one do you think was the most effective? Why?		Answered = 94 (Epipen: 41, Flomax: 34, Detrol: 19). Question skipped = 6	
As a student, how do you perceive DTCAs? Please add specific comments.		Answered = 99, Question skipped = 1	

^a^ Agreement = strongly agree + agree. ^b^ Disagreement = strongly disagree + disagree. 100 out of 211 students enrolled in the course completed the survey.

**Table 3:** Summary of themes identified and representative comments on students’ attitudes and perceptions about the advertisement-based teaching tool.

Theme Identified	Representative Comments
Visual aid	“Because with so many drug names that are similar, is it nice to be able to recall it with a visual such as a commercial.” “I am a visual learner, so if I watch something I am learning about, I remember it better.”
	“These commercials were very popular when they were on tv and it was easy and interesting to make the connection to what we’re learning at the time.” “I thought the commercial made the information stick better for me.”
Connection of drug name to pharmacology and disease state	“The EPIPEN commercial explained indication and instructions for use adequately, which should help to further understanding for the pharmacological mechanisms it is involved in.” “It allowed me to remember that Detrol works on the bladder.” “It helped me associate the medication with urinary retention, making it easier to remember the mechanism.” “It helps with remembering brand and generic names and also major side effects.”
Increased class engagement and retention	“It is an excellent way to keep the class engaged.” “I believe using a quick commercial before teaching about a drug paints an image for students which makes the product easier to relate to and understand its uses.” “There was a question on the exam where I was like: Oh yeah I remember this drug from the commercial video we watched in class” and that helped me pick the right answer so I think you should use commercials in class.”
Patient counseling aid	“What commercials we see are ones that our patients will see, and by seeing them you can understand the draw that they present to patients and why they would want to take the medications and clear up any misconceptions.” “A lot of patients come with questions about the drug commercials they saw on tv and want their doctors to switch their medication just due to popularity.” “At this point in my career, I always view drug commercials as an opportunity to study. When with friends that are not in school for pharmacy, I always use the opportunity to explain more about the drug than the public would be aware of.” “By understanding a drug class and its MOA you understand why side effects occur and while it may scare off a lay person, you can educate people on how to manage these effects, when they are serious and should be reported, and to advocate for what is best for the patient.”

## Data Availability

Survey data were collected using an online survey administered using Survey monkey^®^ (San Mateo, CA, USA). Results will be shared by the author upon request.
